# Comparative outcomes of anatomic and reverse total shoulder arthroplasty for rotator cuff-intact glenohumeral osteoarthritis: a propensity score–matched, age-stratified exploratory analysis

**DOI:** 10.1016/j.jsea.2026.100050

**Published:** 2026-06-24

**Authors:** Chang Hee Baek, Chaemoon Lim, Jung Gon Kim, Bo Taek Kim, Seung Jin Kim

**Affiliations:** Department of Orthopaedic Surgery, Yeosu Baek Hospital, Yeosu, Republic of Korea

**Keywords:** Anatomic total shoulder arthroplasty, Reverse total shoulder arthroplasty, Glenohumeral osteoarthritis, Rotator cuff, Age, Propensity score matching

## Abstract

**Background:**

The optimal implant selection for patients with rotator cuff-intact primary glenohumeral osteoarthritis remains controversial, particularly with respect to patient age. Although reverse total shoulder arthroplasty (rTSA) has been increasingly utilized in older patients, it is unclear whether chronological age alone modifies the relative effectiveness of anatomic total shoulder arthroplasty (aTSA) versus rTSA when baseline characteristics are adequately controlled.

**Methods:**

This retrospective comparative study included patients with rotator cuff-intact primary glenohumeral osteoarthritis who underwent aTSA or rTSA with a minimum follow-up of 3 years. Propensity score matching was performed in a 1:1 ratio to minimize baseline differences between groups, including age, sex, pre-operative clinical outcomes, range of motion, and rotator cuff muscle quality assessed by fatty infiltration. Clinical outcomes were compared using the American Shoulder and Elbow Surgeons (ASES) score as the primary outcome measure. Secondary outcomes included the Constant score, Single Assessment Numeric Evaluation, and post-operative range of motion (forward elevation, external rotation, and internal rotation), as well as complications including scapular notching and acromial or scapular spine stress fracture in the rTSA group and glenoid loosening or rotator cuff failure in the aTSA group. Age-based subgroup analyses (≤70 years vs. > 70 years) and age–procedure interaction analyses were conducted to evaluate whether age modified the association between implant type and outcomes.

**Results:**

After matching, 44 well-balanced pairs (88 patients) were identified. There were no significant differences between the aTSA and rTSA groups in post-operative ASES scores or secondary clinical outcomes. Age-stratified analyses demonstrated consistent results across both age groups, and no significant age–procedure interaction effects were observed. From a clinical perspective, both procedures achieved post-operative ASES and Constant scores exceeding established minimal clinically important difference thresholds. Although the overall complication rate was numerically higher in the rTSA group, these events were predominantly procedure-specific and were largely managed with conservative treatment. Revision rates were low and comparable between groups.

**Conclusion:**

When baseline characteristics are adequately balanced, aTSA and rTSA provide comparable short-term to midterm clinical outcomes in patients with rotator cuff-intact primary glenohumeral osteoarthritis. Chronological age alone does not appear to modify the relative effectiveness of implant type, suggesting that implant selection should be guided by rotator cuff integrity, glenoid morphology, and patient-specific functional demands rather than age alone.

Anatomic total shoulder arthroplasty (aTSA) has long been considered the standard surgical treatment for patients with primary glenohumeral osteoarthritis and an intact rotator cuff, based on its ability to restore native shoulder biomechanics and preserve rotator cuff function.^2^ However, concerns regarding late rotator cuff failure, glenoid component loosening, and revision surgery have prompted increasing interest in reverse total shoulder arthroplasty (rTSA), even in patients without rotator cuff deficiency.^2^

With advances in implant design and surgical technique, rTSA has demonstrated reliable pain relief and functional improvement across a broad range of indications.^11^ As a result, the use of rTSA has expanded beyond cuff tear arthropathy to include selected patients with rotator cuff-intact glenohumeral osteoarthritis, particularly in older individuals.^13^ Several recent studies have reported comparable short-term to midterm clinical outcomes between aTSA and rTSA in this population, with some suggesting lower complication or revision rates following rTSA.^15^ Nevertheless, the optimal implant selection for patients with rotator cuff-intact osteoarthritis remains controversial.

Age is frequently cited as a key factor influencing the choice between aTSA and rTSA. Younger patients are often preferentially treated with aTSA to preserve rotator cuff function and maximize rotational range of motion, whereas rTSA is increasingly favored in older patients due to concerns regarding rotator cuff durability and implant longevity.^12^ Despite this age-based paradigm, limited evidence exists to support a clear age threshold at which one implant type provides superior outcomes over the other.^9^ Moreover, comparisons between aTSA and rTSA are often confounded by baseline differences in patient characteristics, comorbidities, and subtle variations in rotator cuff quality.^3^^,^^6^^,^^8^

To date, few studies have directly evaluated the comparative appropriateness of aTSA and rTSA in patients with rotator cuff-intact osteoarthritis while adequately controlling baseline differences between treatment groups. In particular, it remains unclear whether patient age modifies the relative effectiveness of aTSA versus rTSA when comparable patients are analyzed.

Therefore, the purpose of this study was to compare clinical outcomes between aTSA and rTSA in patients with cuff-intact glenohumeral osteoarthritis using propensity score matching (PSM) to minimize selection bias. A secondary objective was to evaluate whether age modifies the relationship between implant type and clinical outcomes by comparing results in patients aged ≤70 years and >70 years. We hypothesized that, after adjustment for baseline characteristics, clinical outcomes following aTSA and rTSA would be comparable across age groups and that chronological age alone would not be sufficient to determine implant superiority.

## Materials and methods

This retrospective cohort study received approval from the institutional review board (IRB no. P01-2025-3136-001). Due to its retrospective design, the need for written informed consent was waived. All procedures were carried out in accordance with the principles outlined in the Declaration of Helsinki.

### Patient selection

This study included the patients who underwent aTSA or rTSA for primary glenohumeral osteoarthritis with an intact rotator cuff between January 2015 and February 2022. The inclusion criteria were as follows: (1) symptomatic primary glenohumeral osteoarthritis with severe shoulder pain and dysfunction that interferes with daily activities, (2) failure of conservative treatment, (3) concentric or minimally eccentric glenoid wear classified as Walch type^16^ A1 or B1, (4) little or no advanced fatty infiltration in the rotator cuff muscle (Goutallier classification^5^ grade I or II, and (5) no neurologic deficits or infectious diseases. Exclusion criteria were as follows: (1) follow-up < 3 years and (2) unavailable clinical or radiological data.

Implant selection was based on surgeon judgment considering multiple patient-specific factors, including age, functional demand, rotator cuff quality, and glenoid morphology. During the latter part of the study period, the use of rTSA gradually increased in selected elderly patients in response to the growing body of evidence supporting its use in rotator cuff-intact glenohumeral osteoarthritis. The final decision regarding implant type was made after consideration of all clinical and radiographic findings.

### Propensity score matching

PSM was performed to minimize baseline differences between patients undergoing aTSA and rTSA. Propensity scores were estimated using a multivariable logistic regression model that included age, sex, body mass index, follow-up periods, pre-operative forward elevation (FE), pre-operative American Shoulder and Elbow Surgeons (ASES) score, and pre-operative fatty infiltration grades of the rotator cuff muscles (subscapularis, supraspinatus, infraspinatus, and teres minor). One-to-one nearest-neighbor matching without replacement was performed using the estimated propensity scores to create balanced cohorts for comparison. Covariate balance after matching was assessed using standardized mean differences, with values less than 0.10 indicating adequate balance.

### Surgical technique

All procedures were performed by a single experienced surgeon (C.H.B.). After induction of general anesthesia with an interscalene nerve block, the patients were placed in the beach chair position.

### Anatomic total shoulder arthroplasty procedure

aTSA was performed using commercially available anatomic shoulder systems, including the Equinoxe system (Exactech, Gainesville, FL, USA), the Comprehensive Shoulder System (Zimmer Biomet, Warsaw, IN, USA), and the Global Advantage/Global AP system (DePuy Synthes, Warsaw, IN, USA). All procedures were performed through a standard deltopectoral approach. Following humeral head resection, the humeral canal was prepared for implantation of the humeral component. The glenoid was exposed and prepared for implantation of a polyethylene glenoid component. Final component positioning was determined to optimize joint stability and range of motion.

### Reverse total shoulder arthroplasty procedure

rTSA was performed using commercially available reverse shoulder systems, including the Equinoxe Reverse system (Exactech, Gainesville, FL, USA), the Comprehensive Reverse System (Zimmer Biomet, Warsaw, IN, USA), and the Delta Xtend system (DePuy Synthes, Warsaw, IN, USA), according to surgeon preference and implant availability. All procedures were conducted through a standard deltopectoral approach. The cephalic vein and surrounding neurovascular structures were protected. The long head of the biceps tendon was managed with tenotomy or tenodesis, and the subscapularis tendon was assessed and managed based on intraoperative findings. The humeral head was resected at the planned inclination and version, and the humeral canal was prepared for press-fit fixation of the humeral stem. Glenoid preparation included placement of the baseplate, with slight inferior offset when feasible to reduce the risk of scapular notching, followed by glenosphere implantation. Stability and range of motion were confirmed after component implantation. Post-operative rehabilitation followed a standardized protocol with abduction brace immobilization for 6 weeks, progressive passive and active range-of-motion exercises, and subsequent strengthening based on clinical recovery.

### Clinical assessments

Clinical evaluation encompassed pain assessment, patient-reported outcome measures (PROMs), and active range of motion (aROM), with the ASES score defined as the primary outcome measure. Pain levels were recorded using the visual analog scale (VAS) score. PROMs included the ASES score, Constant score, and the Single Assessment Numeric Evaluation (SANE) score. The minimal clinically important difference (MCID) thresholds were defined as 10.3 points for the ASES^10^ score and 8.0 points for the Constant^14^ score. The substantial clinical benefit (SCB) thresholds were defined as 31.5 points for the ASES^10^ score and 19.0 points for the Constant^10^ score. aROM was measured using a goniometer in 2 directions: FE, external rotation (ER) at 0° of abduction. Internal rotation (IR) was graded by noting the highest vertebral level the thumb could reach behind the back, with scores assigned as follows: greater trochanter (0), buttock (2), lumbosacral area (4), L3 (6), T12 (8), and T7 (10). These evaluations were carried out before surgery and at 3 months, 6 months, and 12 months post-operatively, with subsequent follow-ups every 6 months. All evaluations were conducted by an independent research coordinator (S.J.K.) who was blinded to the patient group and surgical procedure.

### Complications

All complications that occurred during the follow-up period were recorded. Complications in the aTSA group included instability or dislocation, implant loosening, and post-operative rotator cuff failure. In the rTSA group, complications included instability or dislocation, acromial or scapular spine stress fractures, and scapular notching. Acromial and scapular spine stress fractures were diagnosed based on clinical symptoms and confirmed using plain radiographs and/or ultrasonography. Scapular notching was evaluated on serial post-operative radiographs obtained during routine follow-up visits and graded according to established radiographic criteria. Implant loosening was defined radiographically by progressive radiolucent lines or component migration observed on serial imaging. All radiological evaluations were independently performed by a musculoskeletal radiologist who was blinded to the patients' clinical information.

### Statistical analysis

Pre-operative and post-operative continuous variables were compared using the nonparametric Wilcoxon signed-rank test. Continuous clinical and radiological outcomes between the 2 groups were compared using the nonparametric Mann–Whitney *U* test. Categorical variables were compared using Fisher exact test.

To explore whether patient age modified the association between implant type and clinical outcomes, an age-based subgroup and interaction analysis was performed within the propensity score–matched cohort. Patients were stratified into 2 age groups using a prespecified threshold of 70 years (≤70 years and >70 years). Clinical outcomes were summarized within each age subgroup according to implant type (aTSA vs. rTSA). Interaction effects between age group and implant type were assessed for continuous outcomes, including ASES score, Constant score, SANE score, FE, ER, and IR, using two-way analysis of variance. The statistical significance of the interaction term was used to determine whether the association between implant type and clinical outcomes differed according to age group. Because these subgroup and interaction analyses were exploratory and potentially underpowered, results were interpreted with caution.

All statistical analyses were performed using the Statistical Package for the Social Sciences (version 21.0; IBM Corp., Armonk, NY, USA). Statistical significance was defined as a two-sided *P* value of <.05.

## Results

### Propensity score matching

**Figure 1:**
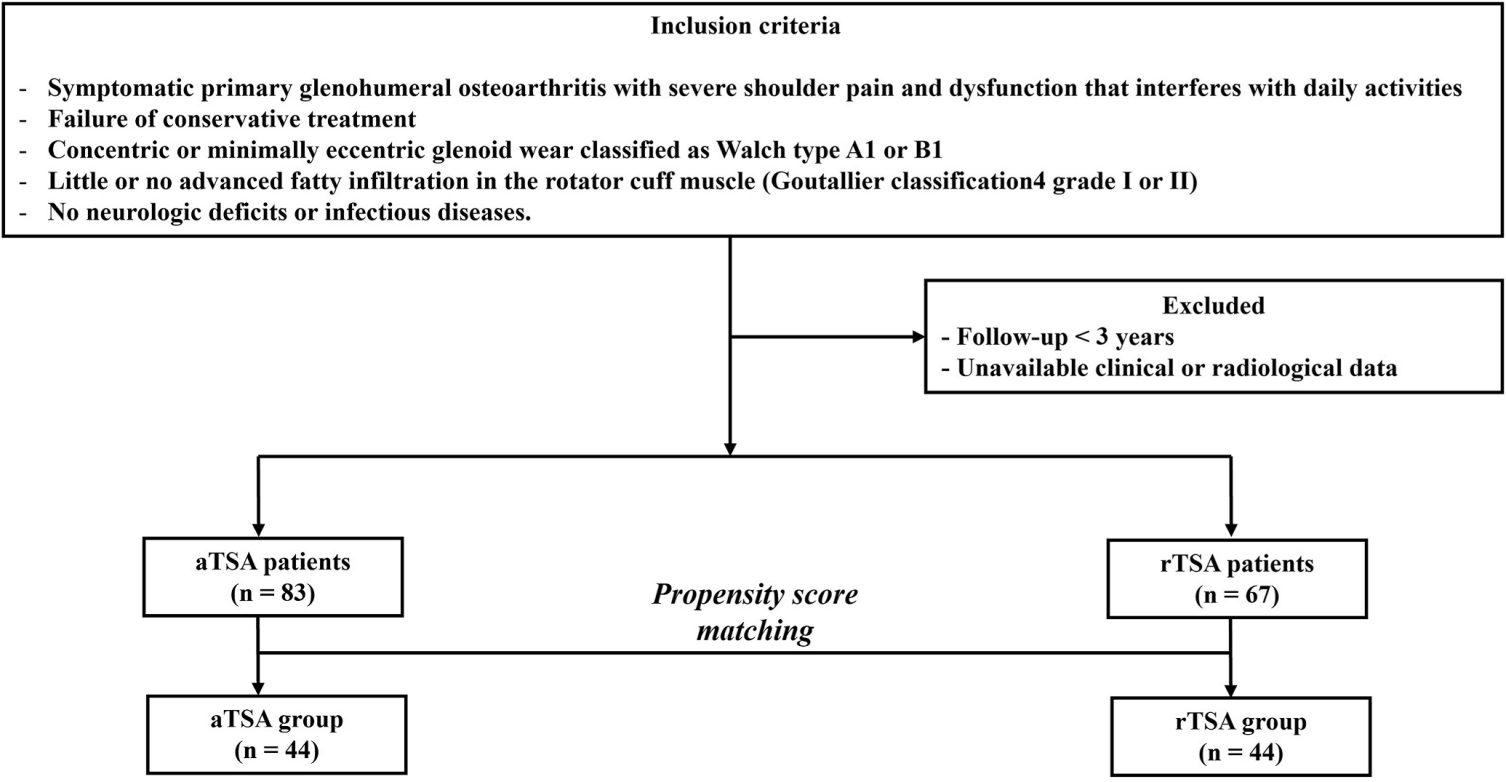
Flowchart for patients. *aTSA*, anatomic total shoulder arthroplasty; *rTSA*, reverse total shoulder arthroplasty.

A total of 168 patients met the inclusion criteria. After excluding 12 patients with a follow-up duration of less than 3 years and 6 patients with unavailable clinical or radiological data, 150 patients (aTSA, n = 83; rTSA, n = 67) were included in the final analysis. Before the matching process, significant differences were observed between the aTSA and rTSA groups in several variables used for matching, as summarized in Table I. PSM was performed in a 1:1 ratio to minimize baseline differences between groups. After matching, 44 matched pairs were identified. The matched cohort included 19 pairs of patients aged ≤70 years and 25 pairs aged >70 years (Fig. 1). Baseline demographic characteristics, pre-operative clinical variables, and rotator cuff muscle quality were well balanced between the anatomic TSA and reverse TSA groups after matching, with no clinically meaningful differences observed (Table II).

**Table I:** Demographic and clinical characteristics of patients before propensity score matching.

Variables	aTSA	rTSA	*P* value
Number of patients	83	67	N/A
Age, mean ± SD, yr	67.0 ± 8.7	73.1 ± 7.2	<.001
Female, %	64 (77.1)	59 (88.1)	.335
BMI, mean ± SD, kg/m^2^	23.8 ± 2.7	23.8 ± 3.8	.815
Arm dominance, n, %	52 (62.7)	41 (61.2)	1.000
Smoking, n, %	4 (4.8)	2 (3.0)	.904
Diabetes mellitus, n, %	13 (15.7)	13 (12.2)	.520
Previous cuff repair, n, %	9 (10.8)	13 (19.4)	.149
Walch class, n, %			.504
A1	66 (79.5)	56 (83.6)	
B1	17 (20.5)	11 (16.4)	
Rotator cuff FI grade			
SSC FI grade, mean ± SD	1.5 ± 0.7	1.5 ± 0.5	.813
SSP FI grade, mean ± SD	1.8 ± 0.6	1.8 ± 0.4	.831
ISP FI grade, mean ± SD	1.6 ± 0.5	1.9 ± 0.3	<.001
Tm FI grade, mean ± SD	1.1 ± 0.3	1.2 ± 0.3	.051
Pre-operative FE ˚, mean ± SD	103.4 ± 19.3°	104.3 ± 34.3°	.010
Pre-operative ASES, mean ± SD	48.1 ± 9.5	47.0 ± 9.4	.010
Mean f/u period, months, mean ± SD (range)	50.1 ± 11.3 (36-75)	53.6 ± 11.4 (36-73)	.036

*aTSA*, anatomic total shoulder arthroplasty; *rTSA*, reverse total shoulder arthroplasty; *SD*, standard deviation; *BMI*, body mass index; *FI*, fatty infiltration; *SSC*, subscapularis; *SSP*, supraspinatus; *ISP*, infraspinatus; *Tm*, teres minor; *FE*, forward elevation; *ASES*, American Shoulder and Elbow Surgeons.

**Table II:** Demographic and clinical characteristics of patients after propensity score matching.

Variables	aTSA	rTSA	*P* value
Number of patients	44	44	
Age, mean ± SD, yr	70.8 ± 6.5	71.3 ± 7.2	.825
Female, %	30 (66.0)	32 (72.7)	.816
BMI, mean ± SD, kg/m^2^	23.8 ± 2.7	23.8 ± 3.8	.835
Arm dominance, n, %	25 (56.8)	24 (55.5)	.111
Smoking, n, %	2 (4.5)	1 (2.3)	1.000
Diabetes mellitus, n, %	8 (18.2)	9 (20.5)	.792
Previous cuff repair, n, %	4 (9.3)	6 (13.6)	.739
Walch class, n (%)			.783
A1	35 (79.5)	37 (84.1)	
B1	9 (20.5)	7 (15.9)	
Rotator cuff FI grade			
SSC FI grade, mean ± SD	1.6 ± 0.6	1.4 ± 0.5	.253
SSP FI grade, mean ± SD	1.8 ± 0.5	1.8 ± 0.4	.904
ISP FI grade, mean ± SD	1.8 ± 0.5	1.8 ± 0.4	.904
Tm FI grade, mean ± SD	1.1 ± 0.4	1.1 ± 0.3	.722
Pre-operative FE ˚, mean ± SD	102.9 ± 18.3	102.5 ± 26.1	.551
Pre-operative ASES, mean ± SD	44.5 ± 7.9	44.5 ± 6.4	.710
Implant type, n (%)			
Eqiunoxe (Exactech)	10 (22.7)	12 (27.3)	
CRS (Biomet)	13 (29.5)	14 (31.8)	
Delta Xtend (Depuy)	21 (47.7)	18 (40.9)	
Mean f/u period, months, mean ± SD (range)	51.7 ± 11.7 (36-72)	49.9 ± 10.1 (36-73)	.583

*aTSA*, anatomic total shoulder arthroplasty; *rTSA*, reverse total shoulder arthroplasty; *SD*, standard deviation; *BMI*, body mass index; *SSC*, subscapularis; *SSP*, supraspinatus; *ISP*, infraspinatus; *Tm*, teres minor; *FE*, forward elevation; *ASES*, American Shoulder and Elbow Surgeons.

### Overall comparison: anatomic total shoulder arthroplasty vs. reverse total shoulder arthroplasty

In the propensity score–matched cohort, both aTSA and rTSA resulted in significant improvements in pain, PROM, and aROM from pre-operative to post-operative assessment (Table III). The primary outcome, the post-operative ASES score, did not differ significantly between the aTSA and rTSA groups. Moreover, no statistically significant difference was observed between the 2 groups with respect to post-operative VAS pain score, Constant score, SANE score, FE, ER, and IR. The mean post-operative ASES and Constant scores in both groups exceeded established MCID thresholds. The SCB defined success rates for ASES and Constant scores were high and comparable between groups, with no statistically significant differences in the proportion of patients achieving SCB for either outcome (ASES SCB: 77.3% vs. 79.5%, *P* = 1.000; Constant SCB: 86.4% vs. 90.9%, *P* = .739). The overall complication rate was higher in the rTSA group; however, this difference did not reach statistical significance (18.2% vs. 4.5%), although this difference did not reach statistical significance (*P* = .089). In the aTSA group, complications consisted of implant loosening in 2 patients and post-operative rotator cuff failure in 1 patient, the latter of which necessitated revision surgery. Post-operative rotator cuff failure was defined as the presence of a new full-thickness rotator cuff tear confirmed by ultrasonography or magnetic resonance imaging in a previously intact rotator cuff, accompanied by relevant clinical symptoms. In the rTSA group, complications included acromial or scapular spine stress fractures in 4 patients, scapular notching in 3 patients, and instability in 1 patient, which required revision surgery. Revision surgery rates were low and identical between groups (2.3% vs. 2.3%, *P* = 1.000).

**Table III:** Comparisons of clinical outcomes between the 2 surgical groups.

Variables	aTSA (n = 44)	rTSA (n = 44)	*P* value
VAS score			
Pre-operative	5.1 ± 1.3	5.3 ± 1.3	.600
Post-operative	1.5 ± 1.3	1.8 ± 1.0	.155
*P* value	<.001	<.001	
ASES score			
Pre-operative	44.5 ± 7.9	44.5 ± 6.5	.710
Post-operative	81.5 ± 7.1	80.7 ± 7.1	.467
*P* value	<.001	<.001	
Constant score			
Pre-operative	46.0 ± 6.1	44.8 ± 7.2	.374
Post-operative	72.0 ± 8.2	72.6 ± 8.7	.917
*P* value	<.001	<.001	
SANE score			
Pre-operative	45.0 ± 7.0	45.7 ± 9.2	.877
Post-operative	71.2 ± 5.3	72.2 ± 8.8	.511
*P* value	<.001	<.001	
MCID for ASES			1.000
Success, n (%)	44 (100/0)	44 (100/0)	
Failure, n (%)	0 (0.0)	0 (0.0)	
SCB for ASES			1.000
Success, n (%)	34 (77.3)	35 (79.5)	
Failure, n (%)	10 (22.7)	9 (20.5)	
MCID for Constant			1.000
Success, n (%)	43 (97.7)	44 (100.0)	
Failure, n (%)	1 (2.3)	0 (0.0)	
SCB for Constant			.739
Success, n (%)	38 (86.4)	40 (90.9)	
Failure, n (%)	6 (13.6)	4 (9.1)	
FE, ˚			
Pre-operative	102.9 ± 18.3	102.5 ± 26.1	.511
Post-operative	148.9 ± 18.2	146.8 ± 18.5	.585
*P* value	<.001	<.001	
ER at 0° of abduction, ˚	<0.001		
Pre-operative	37.1 ± 9.9	34.9 ± 13.1	.170
Post-operative	49.9 ± 11.9	49.7 ± 13.3	.986
*P* value	<.001	<.001	
IR at back			
Pre-operative	4.1 ± 1.5	4.0 ± 1.5	.763
Post-operative	6.5 ± 1.6	6.7 ± 1.7	.752
*P* value	<.001	<.001	
Complications	2 (4.5)	8 (18.2)	.089
Revision surgery	1 (2.3)	1 (2.3)	1.000

*aTSA*, anatomic total shoulder arthroplasty; *rTSA*, reverse total shoulder arthroplasty; *VAS*, visual analog scale; *ASES*, American Shoulder and Elbow Surgeons; *SANE*, Single Assessment Numeric Evaluation; *FE*, forward elevation; *ER*, external rotation; *IR*, internal rotation; *MCID*, minimal clinically important difference; *SCB*, substantial clinical benefit.

Significant *P* value is < 0.05.

### Outcomes in patients aged ≤70 years

Among patients aged ≤70 years, both aTSA and rTSA demonstrated significant post-operative improvements in pain, patient-reported outcomes, and aROM (Table IV). Consistent with the overall analysis, the primary outcome (post-operative ASES score) and all secondary outcomes were comparable between groups, with no statistically significant differences observed. SCB-defined success rates for ASES and Constant scores were similarly high in both groups, and complication and revision rates did not differ significantly.

**Table IV:** Comparisons of clinical outcomes between the 2 surgical groups in patients aged ≤70 y.

Variables	aTSA (n = 19)	rTSA (n = 19)	*P* value
VAS score			
Pre-operative	5.4 ± 1.1	5.2 ± 1.4	.583
Post-operative	1.5 ± 0.9	1.7 ± 1.1	.686
*P* value	<.001	<.001	
ASES score			
Pre-operative	44.5 ± 7.9	44.5 ± 6.2	.817
Post-operative	81.5 ± 7.1	80.7 ± 7.1	.624
*P* value	<.001	<.001	
Constant score			
Pre-operative	44.4 ± 5.9	44.5 ± 8.9	.977
Post-operative	72.5 ± 7.1	73.5 ± 8.5	.751
*P* value	<.001	<.001	
SANE score			
Pre-operative	44.7 ± 6.7	45.6 ± 10.0	.863
Post-operative	71.2 ± 5.3	71.5 ± 6.5	.817
*P* value	<.001	<.001	
MCID for ASES			1.000
Success, n (%)	19 (100.0)	19 (100.0)	
Failure, n (%)	0 (0.0)	0 (0.0)	
SCB for ASES			1.000
Success, n (5)	15 (78.9)	14 (73.7)	
Failure, n (%)	4 (21.1)	5 (26.3)	
MCID for Constant			1.000
Success, n (%)	19 (100.0)	19 (100.0)	
Failure, n (%)	0 (0.0)	0 (0.0)	
SCB for Constant			1.000
Success, n (%)	17 (89.5)	18 (94.7)	
Failure, n (%)	2 (10.5)	1 (5.3)	
FE, ˚			
Pre-operative	100.0 ± 19.2	104.2 ± 30.0	.863
Post-operative	145.8 ± 22.9	147.4 ± 22.3	.908
*P* value	<.001	<.001	
ER at 0° of abduction, ˚			
Pre-operative	37.6 ± 11.6	35.5 ± 13.1	.506
Post-operative	48.7 ± 14.0	47.9 ± 10.2	.863
*P* value	<.001	<.001	
IR at back			
Pre-operative	4.0 ± 1.5	3.8 ± 1.5	.583
Post-operative	6.5 ± 1.7	6.7 ± 1.8	.729
*P* value	<.001	<.001	
Complications	1 (5.3)	3 (15.8)	.609
Revision surgery	0 (0.0)	0 (0.0)	1.000

*aTSA*, anatomic total shoulder arthroplasty; *rTSA*, reverse total shoulder arthroplasty; *VAS*, visual analog scale; *ASES*, American Shoulder and Elbow Surgeons; *SANE*, Single Assessment Numeric Evaluation; *FE*, forward elevation; *ER*, external rotation; *IR*, internal rotation; *MCID*, minimal clinically important difference; *SCB*, substantial clinical benefit.

Significant *P* value is < 0.05.

### Outcomes in patients aged >70 years

In patients aged >70 years, post-operative outcomes following aTSA and rTSA were likewise comparable (Table V). No significant differences were observed in the primary outcome or secondary clinical measures, and both procedures achieved high rates of MCID and SCB for ASES and Constant scores. Although complication rates were numerically higher in the rTSA group, this difference did not reach statistical significance.

**Table V:** Comparisons of clinical outcomes between the 2 surgical groups in patients aged >70 y.

Variables	aTSA (n = 25)	rTSA (n = 25)	*P* value
VAS score			
Pre-operative	5.0 ± 1.1	5.4 ± 1.2	.402
Post-operative	1.5 ± 1.1	1.8 ± 1.0	.110
*P* value	<.001	<.001	
ASES score			
Pre-operative	43.6 ± 7.0	44.5 ± 6.8	.484
Post-operative	80.9 ± 8.0	79.9 ± 8.2	.606
*P* value	<.001	<.001	
Constant score			
Pre-operative	47.3 ± 6.0	45.0 ± 5.7	.127
Post-operative	71.6 ± 9.0	71.9 ± 8.9	.861
*P* value	<.001	<.001	
SANE score			
Pre-operative	45.3 ± 7.4	45.7 ± 8.7	.884
Post-operative	71.4 ± 6.1	72.7 ± 10.3	.453
*P* value	<.001	<.001	
MCID for ASES			1.000
Success, n (%)	25 (100.0)	25 (100.0)	
Failure, n (%)	0 (0.0)	0 (0.0)	
SCB for ASES			.725
Success, n (%)	19 (76.0)	21 (84.0)	
Failure, n (%)	6 (24.0)	4 (16.0)	
MCID for Constant			1.000
Success, n (%)	24 (96.0)	25 (100.0)	
Failure, n (%)	1 (4.0)	0 (0.0)	
SCB for Constant			1.000
Success, n (%)	21 (84.0)	22 (88.0)	
Failure, n (%)	4 (16.0)	3 (12.0)	
FE, ˚			
Pre-operative	105.2 ± 17.6	101.2 ± 23.2	.313
Post-operative	151.2 ± 13.6	146.4 ± 15.5	.202
*P* value	<.001	<.001	
ER at 0° of abduction, ˚			
Pre-operative	36.6 ± 8.6	34.4 ± 13.5	.184
Post-operative	50.8 ± 10.2	51.0 ± 15.3	.992
*P* value	<.001	<.001	
IR at back			
Pre-operative	4.1 ± 1.6	4.1 ± 1.5	.899
Post-operative	6.5 ± 1.6	6.7 ± 1.7	.974
*P* value	<.001	<.001	
Complications	1 (4.0)	5 (20.0)	.202
Revision surgery	1 (4.0)	1 (4.0)	1.000

*aTSA*, anatomic total shoulder arthroplasty; *rTSA*, reverse total shoulder arthroplasty; *VAS*, visual analog scale; *ASES*, American Shoulder and Elbow Surgeons; *SANE*, Single Assessment Numeric Evaluation; *FE*, forward elevation; *ER*, external rotation; *IR*, internal rotation; *MCID*, minimal clinically important difference; *SCB*, substantial clinical benefit.

Significant *P* value is < 0.05.

### Age–procedure interaction analysis

Two-way analysis of variance demonstrated no significant interaction between age group (≤70 years vs. > 70 years) and surgical procedures (aTSA vs. rTSA) for any evaluated outcome measure, including VAS pain score, ASES score, Constant score, SANE score, FE, ER, and IR (all interaction *P* values >0.05) (Table VI). These findings indicate that patient age did not significantly modify the association between implant type and clinical outcomes in the propensity score–matched cohort.

**Table VI:** Interaction effects between age group and surgical procedure.

Variables	F (interaction)	P (interaction)
VAS score	0.03	0.864
ASES score	0.23	0.633
Constant score	0.03	0.864
SANE score	0.03	0.873
FE	0.64	0.425
ER at 0° of abduction	0.03	0.857
IR at back	0.00	0.953

*SANE*, Single Assessment Numeric Evaluation; *VAS*, visual analog scale; *ASES*, American Shoulder and Elbow Surgeons; *FE*, forward elevation; *ER*, external rotation; *IR*, internal rotation; *ANOVA*, analysis of variance.

F-statistics and *P* values are reported for the interaction between age group and surgical procedure based on two-way ANOVA. Statistical significance was set at *P* < .05.

## Discussion

The principal finding of this propensity score–matched, age-stratified analysis is that aTSA and rTSA yielded comparable clinical outcomes in patients with rotator cuff-intact glenohumeral osteoarthritis, with no significant differences observed in the primary outcome measure (ASES score) or in secondary clinical measures. Importantly, chronological age did not modify the relationship between implant type and post-operative outcomes, as evidenced by consistent results across age-based subgroups and the absence of significant age–procedure interaction effects. These findings indicate that, in a carefully selected cohort of patients with preserved rotator cuff integrity, both aTSA and rTSA can provide reliable short-term to midterm functional improvement, and that age alone may not be a sufficient determinant when selecting implant type for the treatment of primary glenohumeral osteoarthritis with an intact rotator cuff.

These findings align with a growing body of literature reporting similar short-term to midterm outcomes between aTSA and rTSA in selected patients with an intact rotator cuff.^3^^,^^6^^,^^8^ Historically, aTSA has been considered the preferred option for cuff-intact osteoarthritis due to its ability to restore native shoulder biomechanics and preserve rotational motion. However, concerns regarding late rotator cuff failure and glenoid loosening have contributed to the expanding use of rTSA, particularly in older patients.^2^ Recent studies have suggested that modern rTSA designs can provide reliable pain relief and functional improvement even in the absence of rotator cuff deficiency, challenging the traditional age-based paradigm for implant selection.^11^^,^^15^

Several recent studies have compared aTSA and rTSA in older patients with preserved rotator cuff integrity and have reported comparable short-term to midterm clinical outcomes between the 2 procedures.^1^^,^^7^ However, many of these studies stratified patients by age without adequately accounting for baseline differences in pre-operative function, comorbidities, or subtle variations in rotator cuff muscle quality, all of which may have influenced implant selection and post-operative outcomes.

A key strength of the present study is the use of PSM to create truly comparable cohorts by controlling not only for age and pre-operative functional status but also for rotator cuff muscle quality as assessed by fatty infiltration. By minimizing these confounders, our analysis allowed for a more direct assessment of whether chronological age independently modifies the relationship between implant type and clinical outcomes. In contrast to traditional age-based paradigms, our findings suggest that chronological age alone, including the commonly used threshold of 70 years, may not be sufficient to guide implant selection between aTSA and rTSA in patients with rotator cuff-intact osteoarthritis. The age threshold of 70 years was selected as a priori because it has been widely used in previous studies and clinical practice as a pragmatic cutoff for implant selection in shoulder arthroplasty.^9^^,^^10^ This threshold is often based on concerns regarding age-related rotator cuff degeneration, reduced tissue quality, and the perceived durability of anatomic implants in older patients.^4^ However, empirical evidence supporting a clear age cutoff remains limited, and the biological heterogeneity among patients of similar chronological age is substantial.^1^

From a clinical relevance standpoint, both procedures achieved post-operative ASES and Constant scores exceeding established MCID thresholds, and high rates of SCB were observed across all age groups. Taken together, these findings suggest that the absence of statistically significant differences between aTSA and rTSA is not merely a consequence of limited statistical power but rather may reflect similar observed outcomes within the limits of this study within the context of patient-perceived functional improvement. Consequently, implant selection should be guided by rotator cuff integrity, glenoid morphology, and patient-specific functional demands rather than chronological age alone.

Moreover, while complication rates were numerically higher in the rTSA group, these events were predominantly procedure-specific and consistent with the known complication profile of reverse shoulder arthroplasty.^17^ The majority of complications in the rTSA group consisted of acromial or scapular spine stress fractures and scapular notching, which were managed successfully with conservative treatment and did not adversely affect overall functional outcomes. Importantly, complications requiring revision surgery were rare and occurred at similarly low rates in both groups. Taken together, these findings suggest that although rTSA may be associated with a higher incidence of certain procedure-related complications, this does not necessarily translate into an increased need for revision surgery or inferior short-term to midterm clinical durability.

Several limitations of this study should be acknowledged. First, all procedures were performed by a single surgeon, which may limit the generalizability of the findings to other surgeons and practice settings. Second, the retrospective design introduces the potential for unmeasured confounding despite the use of PSM. Third, the sample size within age subgroups may have limited the power to detect small age-specific differences, and the subgroup and interaction analyses should therefore be considered exploratory. Therefore, the possibility of a type II error cannot be excluded, and the findings should be interpreted with appropriate caution. Fourth, multiple implant systems were used in both groups, which may introduce heterogeneity; however, this approach reflects real-world clinical practice and enhances the external validity of the findings. In addition, implant selection was not randomized and remained dependent on surgeon judgment, which may have reflected evolving practice patterns during the study period. Therefore, residual selection bias may have persisted despite PSM and adjustment for known confounding variables. Finally, longer-term follow-up is needed to determine whether differences in rotator cuff durability, implant survival, or late complications emerge over time. From a practical perspective, the present findings suggest that implant selection in patients with rotator cuff-intact glenohumeral osteoarthritis should not be based solely on chronological age. Anatomic TSA may remain an appropriate option for patients seeking restoration of native shoulder biomechanics and rotational motion, whereas reverse TSA may be considered in selected patients based on factors such as rotator cuff quality, glenoid morphology, surgeon experience, and individual functional demands. Therefore, treatment decisions should be individualized rather than determined by age alone.

Despite these limitations, this study provides clinically relevant evidence supporting a more nuanced approach to implant selection in cuff-intact glenohumeral osteoarthritis. When baseline characteristics are balanced, aTSA and rTSA offer comparable functional outcomes across age groups, challenging the reliance on age alone as a determinant of implant choice.

## Conclusion

In patients with rotator cuff-intact glenohumeral osteoarthritis, aTSA and rTSA demonstrated similar short-term to midterm clinical outcomes after PSM, with no significant differences observed across age strata. These findings suggest that chronological age alone should not dictate implant selection, and that careful evaluation of rotator cuff integrity and patient-specific factors remain paramount.

## Disclaimers:

Funding: No funding was disclosed by the authors.

Conflicts of interest: The author, their immediate family, and any research foundation with which they are affiliated have not received any financial payments or other benefits from any commercial entity related to the subject of this article.
